# Effect of moderate magnetic fields on the surface tension of aqueous liquids: a reliable assessment†

**DOI:** 10.1039/c9ra00849g

**Published:** 2019-03-29

**Authors:** Masayuki Hayakawa, Jacopo Vialetto, Manos Anyfantakis, Masahiro Takinoue, Sergii Rudiuk, Mathieu Morel, Damien Baigl

**Affiliations:** PASTEUR, Department of Chemistry, École Normale Supérieure, PSL University, Sorbonne Université, CNRS 75005 Paris France damien.baigl@ens.fr; Department of Computer Science, Tokyo Institute of Technology Kanagawa 226-8502 Japan; University of Luxembourg, Physics & Materials Science Research Unit 162a Avenue de la Faiencerie Luxembourg L-1511 Luxembourg

## Abstract

We precisely measure the effect of moderate magnetic field intensity on the surface tension of liquids, by placing pendant drops inside uniform fields where bulk forces due to gradients are eliminated. The surface tension of water is unaffected while that of paramagnetic salt solutions slightly decreases with increasing field strength.

## Abstract 2

A novel setup measures the effect of magnetic field intensities on the surface tension of liquids placed inside uniform fields.

## Graphical abstract

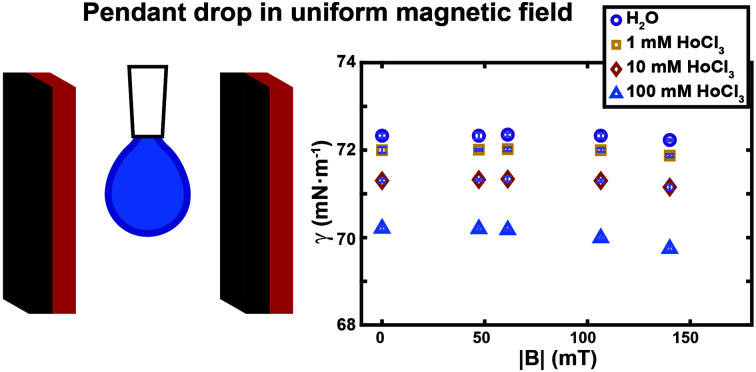

Controlling the properties of fluids in a contactless manner, through the use of external stimuli, offers great advantages in a variety of applications where liquid solutions are involved. Especially, the on-demand variation of their surface/interfacial tension has been proved to be a very efficient method for their handling and manipulation, both in microfluidic channels, or on open surfaces.^1^ For this purpose, various stimulations have been used, such as thermal,^2^ electrical^3^ or optical.^4–9^ In addition, magnetic control has been proved to be suitable for many practical operations, thanks to its various advantages,^10,11^ such as: (i) dropping the need of specific substrate modification, (ii) its relative insensitivity to the variation of solution properties (*e.g.* pH, temperature, ionic concentration, turbidity), (iii) its non-invasive and remote character, and (iv) the good spatio-temporal control. On one hand, fluid manipulation using magnetic fields has been typically performed by adding magnetic particles into the liquids.^12–17^ Despite being a powerful technique, the use of magnetic particles decreases its applicability, increases costs, and causes sample contamination. On the other hand, for complex fluid handling in microfluidic devices, building up surface tension gradients has already been proved to be suitable in controlling various fluidic operations. Even though intense research effort has been put on studying the magnetic effect on the properties and behaviour of water or other liquids,^18–21^ only a few studies on their surface tension were reported.^22–25^ This is true especially for relatively low magnetic fields,^26,27^ as the one that can be produced with small permanent magnets used inside microfluidic chips (∼100 mT).

Up to now, surface tension measurements have always been performed using the pendant drop method, with drops placed in a non-homogeneous magnetic field. The pendant drop is the most commonly used method to determine the interfacial tension of liquid/liquid or liquid/gas interfaces.^28^ It is based on analysing the shape of drops hanging from a capillary, and deducing the surface tension value with a mathematical model which considers that the drop shape is dictated by the interplay of the forces acting on the drop (surface tension and gravity). However, a non-homogeneous magnetic field exerts an additional bulk force on the liquid drop that causes its deformation.^20,29^ Such magnetic force will be attractive if the liquid is paramagnetic (having a magnetic susceptibility *χ* > 0), while it will be repulsive if the liquid is diamagnetic (*χ* < 0). This interaction can deform the pendant drop in a way that is not considered by the software used for analysis. In these conditions, fitting the drop shape does not allow to distinguish between the bulk and the eventual surface effects. With such an approach, only an apparent surface tension can be detected by the instrument.^27,30^ In order to suppress the bulk component, which arises in the presence of a magnetic gradient, we constructed an experimental setup where surface tension measurements were performed using the pendant drop method inside highly uniform magnetic fields. Unlike ferrofluid drops that can elongate along field lines due to their strong magnetic response,^31^ most common liquid drops maintain their shape under uniform fields. Therefore, with our experimental setup, the drop deformation was essentially due to surface effects only and fitting its shape allowed us to accurately extract its surface tension. By varying the field intensity, we could reliably establish the effect of magnetic field on the evolution of surface tension of various diamagnetic and paramagnetic solutions (water and aqueous solutions containing dissolved paramagnetic salts).

Fig. 1 shows the two experimental setups used to measure the surface tension of aqueous solutions by means of the pendant drop technique. Fig. 1A shows a sketch of the configuration used to measure the apparent surface tension of drops in a non-homogeneous magnetic field generated by a small permanent magnet placed below the drop. This geometry is similar to the one commonly reported in literature.^26,27,32^ It has been used in order to compare our experiments with published results, and with the surface tension of the same solutions as measured in a homogeneous magnetic field of similar intensity. Fig. 1B shows the measured map of the magnetic field norm (|*B*|) around a drop in this configuration. Both the components of the magnetic field perpendicular and parallel to the magnet surface were highly non-homogeneous at the drop level (Fig. S1A and B†), resulting in a variation in |*B*| of around 70 mT from the drop neck to its bottom. In order to obtain a magnetic field that is homogeneous over the whole drop volume, a novel setup was developed (Fig. 1C). We placed the drop at a precisely controlled distance between two large-sized parallel magnetic plates facing their opposite poles. It is important to note that permanent magnets of this size, when facing each other at relatively short distances (in the order of centimetres) exert a very strong attractive force. Therefore, a rigid scaffold was constructed in order to keep the two magnets at a controlled distance. This set-up was conceived to be compatible with a KRÜSS tensiometer for surface tension measurements. All components used (*e.g.*, syringes and pipette tips) were non-metallic in order to avoid interactions with the strong magnets. In this way, a drop could be produced in the centre of the two parallel magnet surfaces, where both the perpendicular and parallel components of the magnetic field were highly homogeneous (Fig. S1C and D†). Fig. 1D shows the measured map of |*B*| when the distance between the magnet surfaces and the centre of the drop was *d* = 5 cm. In that case, the |*B*| value around the drop was equal to 141 ± 0.5 mT. Varying *d* allowed us to tune the magnetic field intensity while keeping the field highly homogeneous at the drop region (Fig. S2†).

**Fig. 1:**
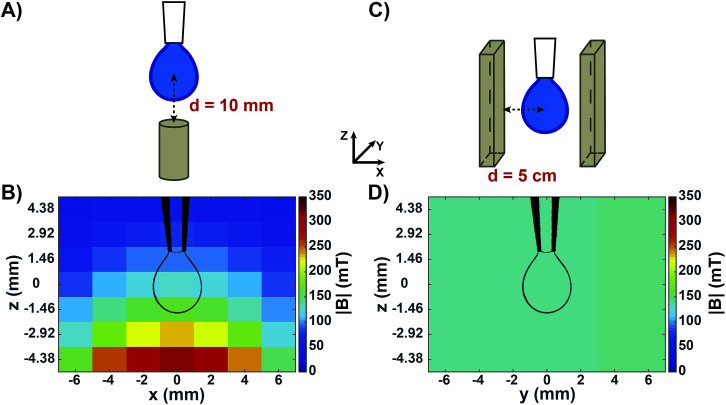
Experimental setups and measured maps of the magnetic field around a pendant drop. (A) Sketch of a pendant drop in a non-homogeneous magnetic field generated by a small permanent magnet placed below the drop. The distance between the magnet surface and the centre of the drop was *d* = 10 mm. (B) Map of the measured magnetic field norm (|*B*|) around the drop in (A). (C) Sketch of a pendant drop in a homogeneous magnetic field generated by two permanent magnets placed parallel to each other, each at a distance *d* = 5 cm from the drop centre. (D) Map of the measured magnetic field norm (|*B*|) around the drop in (C). The indicated *x*, *y*, *z* coordinates refer to all (A–D) panels. Drawings of the drop contours (to scale) are shown in (B) and (D) for helping visualizing the drop position in the magnetic field.

All experiments were conducted at controlled room temperature and humidity (*T* = 22.3 ± 0.4 °C; relative humidity = 31.4 ± 5.8%). For each sample, the air/water surface tension values were obtained by averaging measurements over at least ten different drops. The apparent surface tension (γ*) calculated by the tensiometer software of a diamagnetic and a paramagnetic liquid measured in a non-homogeneous magnetic field are reported in Fig. 2A. The diamagnetic liquid is pure water, having a volumic magnetic susceptibility *χ* = −9.5 ± 0.4 × 10^−6^; the paramagnetic sample is an aqueous HoCl_3_ solution at a concentration of 100 mM, with *χ* = 45 ± 1.7 × 10^−6^ (Fig. S3†). The actual surface tension (*γ*) of those liquids, in the absence of the magnetic field, is reported on the left side of the graph. We note that the addition of HoCl_3_ induced a weak surface tension decrease of the aqueous solution. Approaching a small permanent magnet from the bottom (Fig. 2A, middle) up to a distance *d* = 10 mm (corresponding magnetic field map in Fig. 1B) caused a deformation of the drop as outlined above: a diamagnetic drop was repelled (compressed), whereas a paramagnetic one was attracted (elongated).

**Fig. 2:**
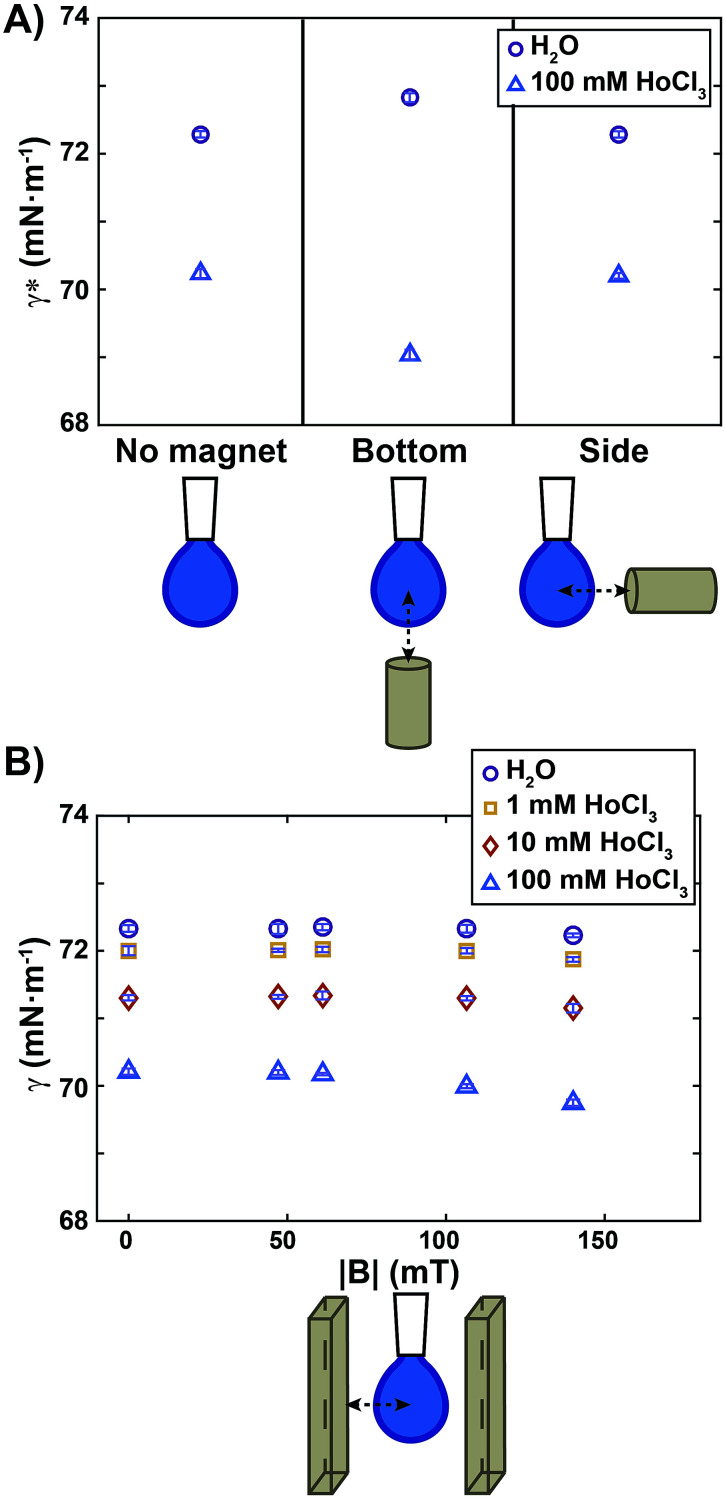
Apparent surface tension (γ*) and surface tension (*γ*) measurements for diamagnetic and paramagnetic liquids. (A) Apparent surface tension (γ*) measurements for water (violet circles) and a solution of 100 mM HoCl_3_ (blue triangles) in the absence of a magnetic field (left), with a small permanent magnet approached from the bottom (centre) at a distance *d* = 10 mm (see Fig. 1 for a map of the corresponding magnetic field), and with a small magnet approached from the side at a distance *d* = 10 mm. Note that γ* in (A) without the magnet (left side), is an actual surface tension value (*γ* = γ*). (B) Surface tension (*γ*) measurements for water (violet circles) and solutions of increasing HoCl_3_ concentration as a function of the norm of the magnetic field at the drop level. The map of the magnetic field in Fig. 1 corresponds to the points at |*B*| = 140 mT. Symbols and error bars show mean ± SD from 10 individual experiments.

Upon fitting of the drop profiles, the software of the instrument detected an apparent surface tension variation that was quantified as Δγ* = 0.5 mN m^−1^ for pure water (violet circles, Fig. 2A), and Δγ* = −1.2 mN m^−1^ for 100 mM HoCl_3_ (blue triangles). Similar Δγ* values in a non-homogeneous magnetic field were reported in ref 27 both for water and a paramagnetic solution (in their case 200 mM aqueous solution of FeCl_3_).

To show that the change in the apparent surface tension, detected for compressed and elongated drops, was due to a bulk magnetic effect and not to a variation of the surface properties of the solutions in the presence of the magnetic field, we placed the magnet at the same distance from the drop, but on its side, so that the magnetic field gradient pointed from the left to the right side of the drop. As a result, the drop was repelled (pure water) or attracted (100 mM HoCl_3_ solution), but no compression or elongation occurred. Consequently, the apparent surface tension detected in this configuration (Fig. 2A, right) was the same as without the magnetic stimulation for both solutions. We thus conclude that a setup where a pendant drop is placed in a non-homogeneous magnetic field is not appropriate for measuring the actual effect of the magnetic field on the surface tension.

To rule out bulk effects that interfere with the measurements, and to quantify the effect of the magnetic field intensity on surface tension, we performed measurements using the setup depicted in Fig. 1C. Fig. 2B shows the surface tension measured when a drop of a diamagnetic or paramagnetic solution is placed inside a homogeneous magnetic field of varied intensities. We quantified the maximum surface tension variation as Δ*γ* = *γ*_0_ − *γ*_140_, where *γ*_0_ and *γ*_140_ were the surface tension without magnetic stimulation, and at the maximum magnetic field intensity used (|*B*| = 140 mT), respectively. For pure water (violet circles in Fig. 2B) we obtained Δ*γ* = −0.1 mN m^−1^, a decrease that was smaller than our experimental error. We can therefore conclude that, under moderate magnetic fields up to 140 mT, no magnetic effect on the surface tension was detected. We do not exclude that significant surface tension variation can occur at higher magnetic field intensities, as reported, for example, in ref. 23 and 25 where a magnetic field two orders of magnitude stronger than ours, (10 T) was employed.

A similar result, Δ*γ* = −0.1 mN m^−1^, was also obtained for diamagnetic aqueous solutions composed of 1 mM and 10 mM HoCl_3_ (*χ* = −8.4 × 10^−6^ and *χ* = −3.4 × 10^−6^, respectively, see Fig. S3†). When a paramagnetic drop composed of 100 mM HoCl_3_ was placed inside the homogeneous magnetic field, the maximum surface tension difference measured was Δ*γ* = −0.46 mN m^−1^. Such amplitude is of similar order of magnitude to that caused by small temperature changes. For instance, the surface tension of water typically varies as Δ*γ* = −0.1 mN m^−1^ K^−1^.^33^ We can therefore conclude that a 100 mM HoCl_3_ paramagnetic solution shows only a very small decrease of its surface tension in a moderate, homogeneous, magnetic field.

## Conclusions

This communication reports a custom-built setup that can be of general interest in characterizing the effect of the magnetic field on the surface/interfacial tension of liquids. The setup allowed us to precisely measure the surface tension of pendant drops placed into a homogeneous magnetic field of moderate intensities, whose strength is comparable to the one commonly used in microfluidic devices and/or for the magnetic handling of drops. We note that, even though not showed, the liquid/liquid interfacial tension of pendant drops immersed in an outer liquid could be measured as well with our configuration. The surface tension of diamagnetic and paramagnetic solutions could be analysed for various magnetic field intensities, and compared to previously reported experiments where the drops were placed in non-homogeneous magnetic fields. Being unaffected by bulk effects, our measurement in homogeneous fields offers a correct and more reliable value. We established that, while pure water was unaffected, only a small negative variation has been detected for the paramagnetic solution tested (holmium chloride dissolved in water) in the range of the moderate magnetic field intensities explored.

## Conflicts of interest

There are no conflicts to declare.

## Supplementary Material

RA-009-C9RA00849G-s001
