# Supplementary material for: Effect of moderate magnetic fields on the surface tension of aqueous liquids: a reliable assessment
Source: RSC Adv. 2019 Mar 29;9(18):10030–3. doi: 10.1039/c9ra00849g (PMC9062369; doi:10.1039/c9ra00849g)
Supplement: RA-009-C9RA00849G-s001 [file RA-009-C9RA00849G-s001.pdf]

Electronic Supplementary Material (ESI) for RSC Advances.
This journal is © The Royal Society of Chemistry 2019

Supporting information for:

Effect of Moderate Magnetic Fields on the Surface Tension of Aqueous Liquids: a Reliable Assessment

Masayuki Hayakawa,[1,2] Jacopo Vialetto,[1] Manos Anyfantakis,[1,3] Masahiro Takinoue,[2] Sergii Rudiuk,[1] Mathieu Morel[1] and Damien Baigl[1]*

[1]*PASTEUR, Department of Chemistry, École Normale Supérieure, PSL University, Sorbonne Université, CNRS, 75005 Paris, France.*

[2]*Department of Computer Science, Tokyo Institute of Technology, Kanagawa 226-8502, Japan.*

[3]*University of Luxembourg, Physics & Materials Science Research Unit, 162a Avenue de la Faiencerie, Luxembourg L-1511, Luxembourg.*

Contents

1) Materials and Methods

2) Supplementary Figures S1-S3

1) Materials and Methods

Materials. Ultrapure MQ water (resistivity: 18.2 MΩ·cm) was used for all the experiments. Holmium chloride (99.9% trace metals basis) was purchased from Sigma-Aldrich and used without any further purification. The small (NdFeB cylinders, 12 mm x 60 mm) and big (NdFeB blocks, 110.6 x 89 x 19.5 mm, approximate strength 1960 N) magnets were purchased from Supermagnete.

Measurements of the magnetic susceptibility of $HoCl_3$ solutions. The volume magnetic susceptibility of aqueous solutions as a function of $HoCl_3$ concentration (Figure S2) was measured using a magnetic susceptibility balance Mark1. For each concentration, the average of three independent measurements was obtained.

Measurements of the magnetic field. Each magnetic field map was measured with a GM08 Gaussmeter (Hirst Magnetic Instruments Ltd.), equipped with a Standard Transverse Hall Probe. The probe was mounted on a linear translation stage (0.01 mm resolution, Edmund Optics). The perpendicular ($B_\perp$) and parallel ($B_\parallel$) components of the magnetic field were measured by placing the probe:

- in the xy ($B_\perp$) and xz ($B_\parallel$) planes in the heterogeneous field configuration (Figures 1A-B and S1A-B)
- in the yz ($B_\perp$) and xz ($B_\parallel$) planes in the homogeneous field configuration (Figures 1 C-D and S1C-D)

The magnetic field norm was calculated as follows: $|\mathbf{B}| = (B_\perp^2 + B_\parallel^2)^{1/2}$.

Experimental setup for homogeneous magnetic field stimulation of pendant drops. The homogeneous magnetic field was generated using a pair of permanent NdFeB magnets (110.6 x 89 x 19.5 mm, Supermagnete). For safety reasons, they were covered with a cushioning material and were placed so that their opposite poles were parallel and faced each other. The distance between them (2*d*) was controlled using a solid spacer, and it was varied by changing the spacer thickness. In the space between the magnets, pendant drops of the solutions were formed using a plastic syringe. To avoid magnetic interactions, we attached a plastic pipette tip to the syringe, instead of using a metallic needle.

Measurement of surface tension of water solutions. The surface tensions of solutions with varying $HoCl_3$ concentrations were measured with a DSA30 Drop Shape Analysis System (Krüss), by fitting the shape of a pendant drop (volume ~ 7 µL) with the Young-Laplace equation. Each measurement was repeated at least ten times. All experiments were conducted at temperature 22.3 ± 0.4 °C and humidity 31.4 ± 5.8 %.

2) Supplementary Figures S1-S3

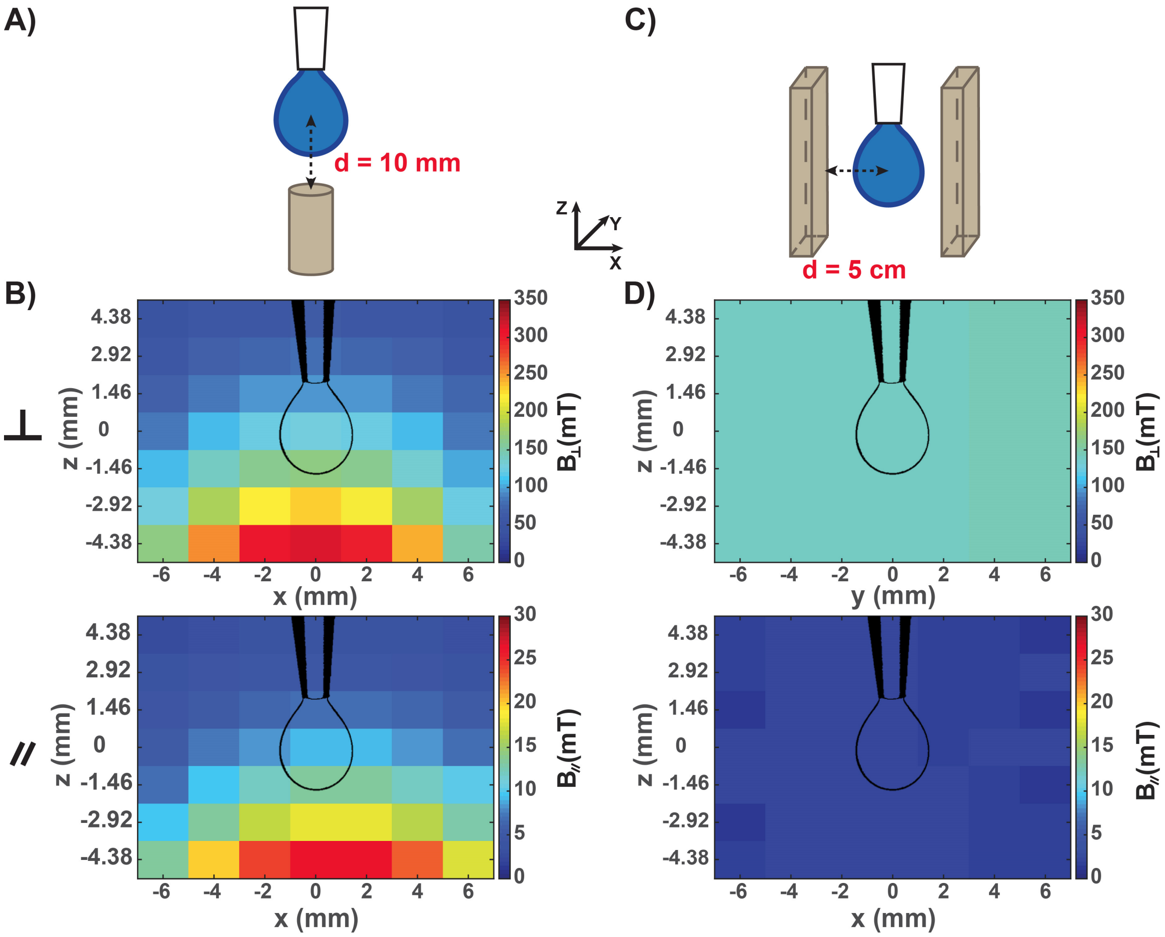

Figure S1. Perpendicular and parallel components of the magnetic field in the two pendent drop configurations of Figure 1. A) Sketch of a pendant drop in a non-homogeneous magnetic field generated by a small permanent magnet placed below the drop. The distance between the magnet surface and the centre of the drop was $d = 10$ mm. B) Map of the measured perpendicular (top) and parallel (bottom) components of the magnetic field around the drop in A). C) Sketch of a pendant drop in a homogeneous magnetic field generated by two permanent magnets placed parallel to each other, each at a distance $d = 5$ cm from the drop centre. D) Map of the measured perpendicular (top) and parallel (bottom) components of the magnetic field around the drop in A). Drawings of the drop contours (to scale) are shown in B) and D) for helping visualizing the drop position in the magnetic field.

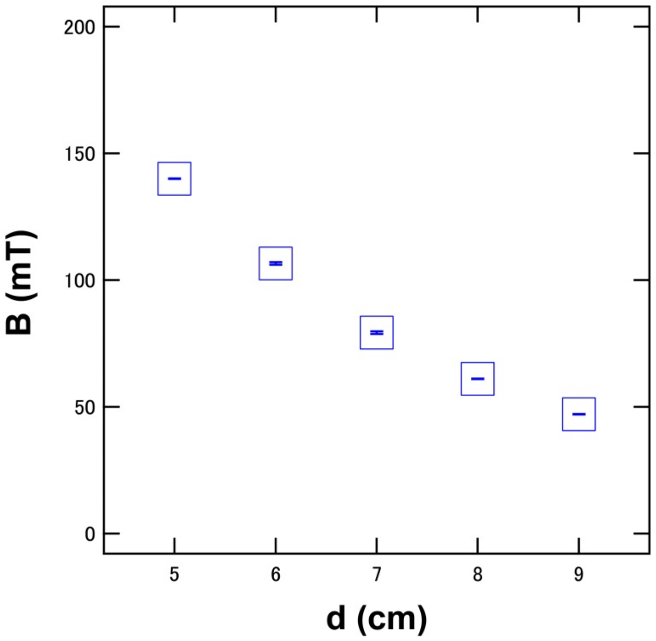

Figure S2. Magnetic field intensity as a function of the parallel magnets surface to drop centre distance (*d* in Figure 1C). Symbols and error bars show mean ± sd from 3 independent measurements. The point at *d* = 5 cm corresponds to the intensity map in Figure 1D.

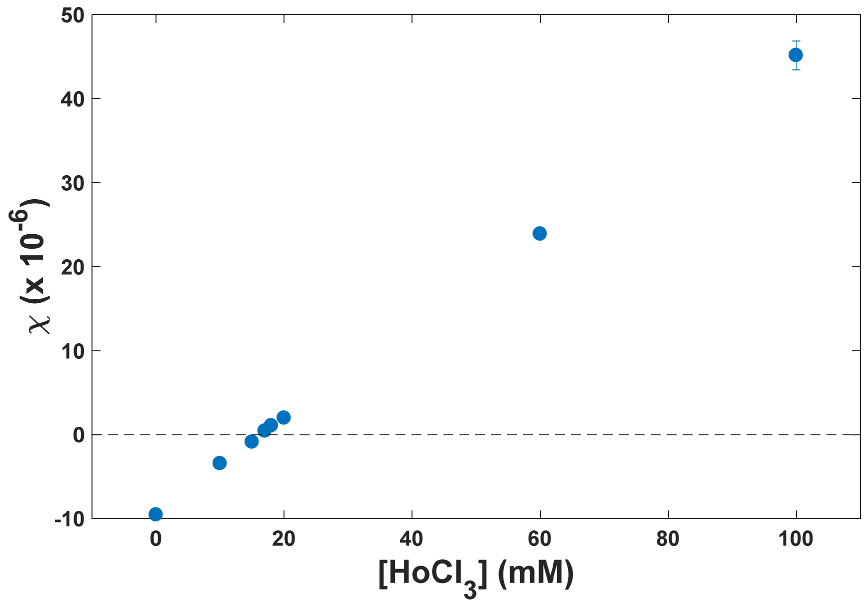

Figure S3. Volume magnetic susceptibility (χ) of aqueous solutions as a function of $HoCl_3$ concentration. Symbols and error bars show mean ± sd from 3 independent measurements.
